# Mesoporous Porphyrin-Silica Nanocomposite as Solid Acid Catalyst for High Yield Synthesis of HMF in Water

**DOI:** 10.3390/molecules26092519

**Published:** 2021-04-26

**Authors:** Arindam Modak, Akshay R. Mankar, Kamal Kishore Pant, Asim Bhaumik

**Affiliations:** 1 Catalytic Reaction Engineering Lab, Department of Chemical Engineering, Indian Institute of Technology Delhi, Delhi 110016, India; arindam_modak_2006@yahoo.co.in (A.M.); akshaymankar79@gmail.com (A.R.M.); 2 School of Materials Sciences, Indian Association for the Cultivation of Science, 2A & 2B Raja S. C. Mullick Road, Jadavpur, Kolkata 700032, India

**Keywords:** mesoporous porphyrin polymer, solid acid catalyst, fructose conversion, microwave synthesis in water, 5-hydroxymethyl furfural (HMF)

## Abstract

Solid acid catalysts occupy a special class in heterogeneous catalysis for their efficiency in eco-friendly conversion of biomass into demanding chemicals. We synthesized porphyrin containing porous organic polymers (PorPOPs) using colloidal silica as a support. Post-modification with chlorosulfonic acid enabled sulfonic acid functionalization, and the resulting material (PorPOPS) showed excellent activity and durability for the conversion of fructose to 5-hydroxymethyl furfural (HMF) in green solvent water. PorPOPS composite was characterized by N_2_ sorption, FTIR, TGA, CHNS, FESEM, TEM and XPS techniques, justifying the successful synthesis of organic networks and the grafting of sulfonic acid sites (5 wt%). Furthermore, a high surface area (260 m^2^/g) and the presence of distinct mesopores of ~15 nm were distinctly different from the porphyrin containing sulfonated porous organic polymer (FePOP-1S). Surprisingly the hybrid PorPOPS showed an excellent yield of HMF (85%) and high selectivity (>90%) in water as compared to microporous pristine-FePOP-1S (yield of HMF = 35%). This research demonstrates the requirement of organic modification on silica surfaces to tailor the activity and selectivity of the catalysts. We foresee that this research may inspire further applications of biomass conversion in water in future environmental research.

## 1. Introduction

The depletion of non-renewable fossil-derived resources is a prime concern for our society. It would be a major breakthrough if we could achieve considerable replacement of fossil fuels by renewable biomass-based feedstock as green fuel [1]. Among biomass-derived platform chemicals, 5-hydroxymethyl furfural (HMF) is very demanding and versatile, since it can be transformed into numerous important chemicals, such as 2,5-furandicarboxylic acid, 2,5-diformylfuran, levulinic acid and 2,5-dimethyl furan, which are precursors for polymers and fuels [2]. HMF was initially produced by dehydration of monosaccharides using homogeneous mineral acids like HCl, H_2_SO_4_ and HNO_3_. However, their high corrosive properties as well as toxicity to the environment has motivated researchers to invent more user-friendly catalytic reactions. Moreover, soluble acids may create side reactions, decrease selectivity of the product, and be difficult to separate from the product mixtures. Commercial Amberlyst-15 shows 80% HMF yield from fructose in DMSO and ionic liquid mixtures [3,4]. Though ionic liquids are explored in the synthesis of HMF from cellulose, their application using a biphasic solvent system, water-methyl isobutyl ketone, water-butanol, and water-THF is disadvantageous due to the issues of recyclability and the toxicity of applying organic solvents [5,6]. Thus, solid-supported catalysis for the synthesis of HMF is quite challenging under environmentally benign reaction conditions. High-density solid acids covalently embedded on a recyclable support [7] may catalyze this conversion processes very efficiently. HMF produced through solid acid catalysts, e.g., AlCl_3_, SnCl_4_, polyoxometalates, WO_3_/SO_3_H-containing zirconia and cation exchange resins, show relatively lower product yield and poor selectivity in water [8,9,10]. Early research carried out with FeCl_3_, either in a batch reaction or using a continuous flow micro-reactor, shows promising advantages for fructose conversion to HMF in high yields [11]. Studies also report that sulfated TiO_2_-ZrO_2_ is an excellent dehydration catalyst that converts glucose or fructose to HMF [12]. Although solid acid catalysts are explored for the dehydration of biomass, high surface area, poor dispersibility and stability, and inadequate covalency between the active sites within the support may be problematic for consecutive recycling purposes. Although ordered mesoporous silica are well explored for one-pot conversion of biomass to HMF, post-functionalization of active sites on the silica support using organosilane are disadvantageous due to self-polymerization among silanes. Furthermore, accessible pores can also be blocked with this approach. In addition, reaction in neat water may be problematic due to poor solubility of HMF and low stability of hydrophilic silica. Under such circumstances, hydrophobic modification is required to tailor the activity and stability of silica supports [13].

Functional porous organic polymers (POPs) have a huge scope in solid catalysis because of their stability and scalability in synthesis as well as their tailorability in porous structures [14,15,16]. POPs possesses abundant surface-exposed aromatic sites for easy functionalization with surface bound Brønsted acids, which facilitates strong interaction with substrates [17]. However, controlling activity and selectivity with polymer-based catalysts remains a significant challenge due to microporosity and inadequate interaction with substrates. Hybrid catalysts are appealing, where inorganic components serve as support, and the organic modification may control diffusion and the product selectivity [18]. Rational design of hybrid catalysts thus remains challenging, particularly while accomplishing the reactions in water [19]. Herein, we report that POPs tethered on colloidal silica enriched with sulfonic acid facilitates fructose conversion in water, while the sulfonated porphyrin polymer marginally produces HMF in water under similar reaction conditions. Although related polymeric solid acids have been recently explored for one-pot conversion of fructose to HMF, poor yields were observed in water [20]. Thus, this research can offer significant opportunities for sustainable development of green catalysts for biomass conversion in water.

## 2. Results

### Synthesis and Characterization of PorPOPS

**Scheme 1:**
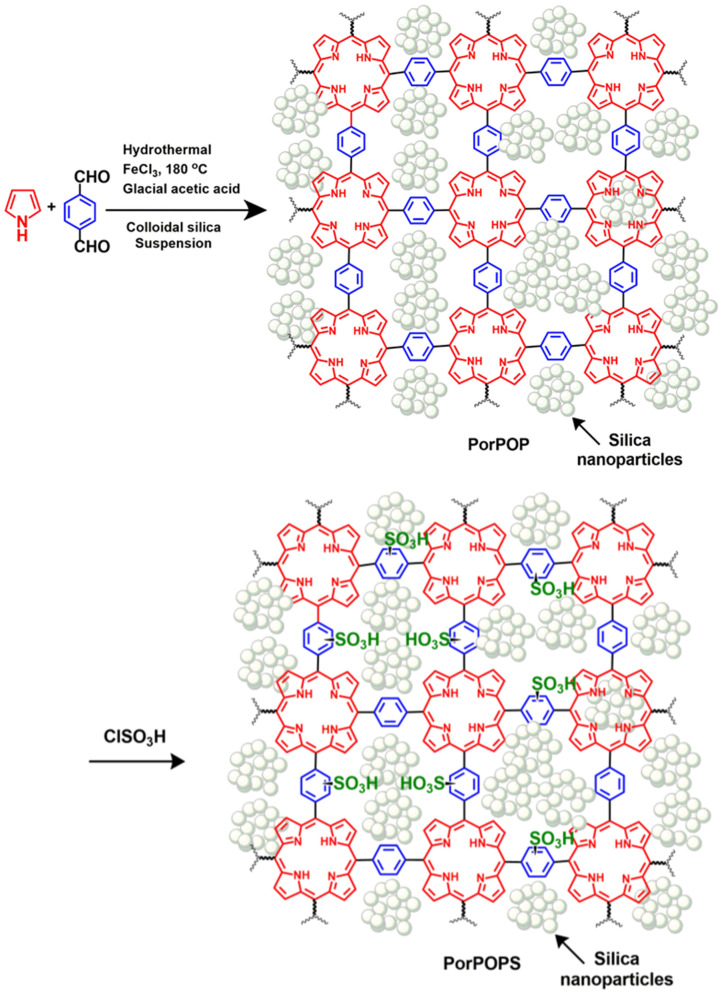
Schematic representation for the synthesis of PorPOPS.

**Figure 1:**
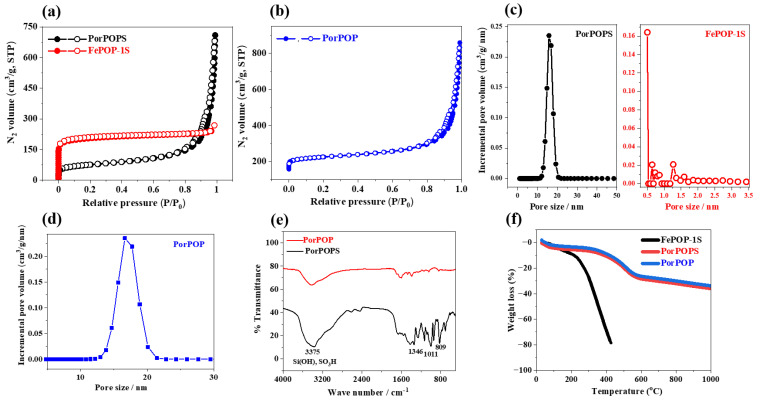
(**a**,**b**) N_2_-sorption isotherm; (**c**,**d**) pore size distribution; (**e**) FTIR-spectra; (**f**) TGA (air) analysis of Fe-POP-1S, PorPOP and PorPOPS.

Hybrid silica-porphyrin composite (PorPOPS) was prepared through a one-pot bottom-up approach using solvothermal condensation polymerization of pyrrole and terephthalaldehyde [21,22]. To functionalize with sulfonic acids, post-functionalization was done with chlorosulfonic acid (ClSO_3_H), as shown in Scheme 1. Initial characterization by FESEM, TEM, N_2_ sorption, and XPS analysis confirms that porphyrin polymers are indeed functionalized by sulfonic acids and are coated with silica nanoparticles. Furthermore, we compared PorPOPS with un-functionalized PorPOP as well as FePOP-1. Porosity and pore size distribution was obtained from N_2_ sorption isotherms, as shown in Figure 1a–d. Both PorPOPS and PorPOP exhibited N_2_ uptake at low P/P_0_, followed by flat interpolation within (0.2–0.8) P/P_0_ and steep uptake beyond 0.8 P/P_0_, corresponding to the mesoporous materials [23]. In contrast, FePOP-1S showed microporosity, with steep uptake of N_2_ at low P/P_0_ (<0.1) and almost linear extrapolation in the entire P/P_0_ region (0.1–0.99). These characteristics indicate that incorporation of silica during synthesis prevents the formation of microporous polymers and introduces extended inter-particle mesoporous materials.

The Brunauer-Emmett-Teller (BET) surface area was calculated to be 260 and 280 m^2^/g for PorPOPS and un-functionalized PorPOP, respectively, which is lower compared to the pristine porphyrin polymer (FePOP-1) (860 m^2^/g) and even the sulfonated FePOP-1S (589 m^2^/g) because of the inclusion of silica nanoparticles that prevents accessible pores [22]. Pore size distribution of PorPOPS and PorPOP from Figure 1c,d clearly signify that the hybrid nanostructure contains distinct mesopores of ~15 nm, which is attributed primarily to the interparticle mesopores. In contrast, sulfonated FePOP-1S possesses small micropores (0.5–1.5 nm). It is noteworthy that, unlike microporous porphyrin, mesoporous hybrid PorPOPS could be advantageous as it obviates the diffusion limitation of substrates imposed by micropores [24]. Thus, with regard to the conversion of large biomass molecules like fructose, the developed organic–inorganic hybrid catalyst demands merit. Nevertheless, pore size has a profound influence on the yield as well as the selectivity of HMF, as shown by Du et al. [25]. Total pore volume was calculated from N_2_ sorption isotherm, and it was found to be 0.98 cm^3^/g. This large pore volume is attributed to the presence of inter-particle mesopores. The FT-IR spectrum is shown in Figure 1e, where a sharp and high intense peak appears at ~3374 cm^−1^, corresponding to the –OH of –SO_3_H and silica particles in PorPOPS.

The existence of a porphyrin ring in PorPOPS is attributed to the presence of bands at 1267 and 1137 cm^−1^, corresponding to the C–N stretching of pyrrole. Subsequently, other bands at 1668 and 1430 cm^−1^ are due to phenyl C=C stretching in the porphyrin ring [26]. Symmetric and asymmetric stretching of C–S and C–SO_3_H were observed at 471, 1075 and 1350 cm^−1^ [27]. Such characteristics are assigned the confirmation of sulfonic acid in PorPOPS, whereas these bands are absent in un-functionalized PorPOP. A TGA plot is shown in Figure 1f; it suggests a stable framework up to 400 °C (weight loss ca. ~10%). Total weight loss of ~37% up to 1000 °C suggests that PorPOPS and PorPOP consists of ~27% organic groups (porphyrin functionality). The sulfonated organic polymer, FePOP-1S, is stable up to 280 °C (weight loss ca. ~10%) and completely collapses above 400 °C. This result suggests that PorPOPS and PorPOP, composed of a hybrid composite containing both porphyrin and silica functionality, have considerably high thermal stability over the porphyrin polymer alone.

**Figure 2:**
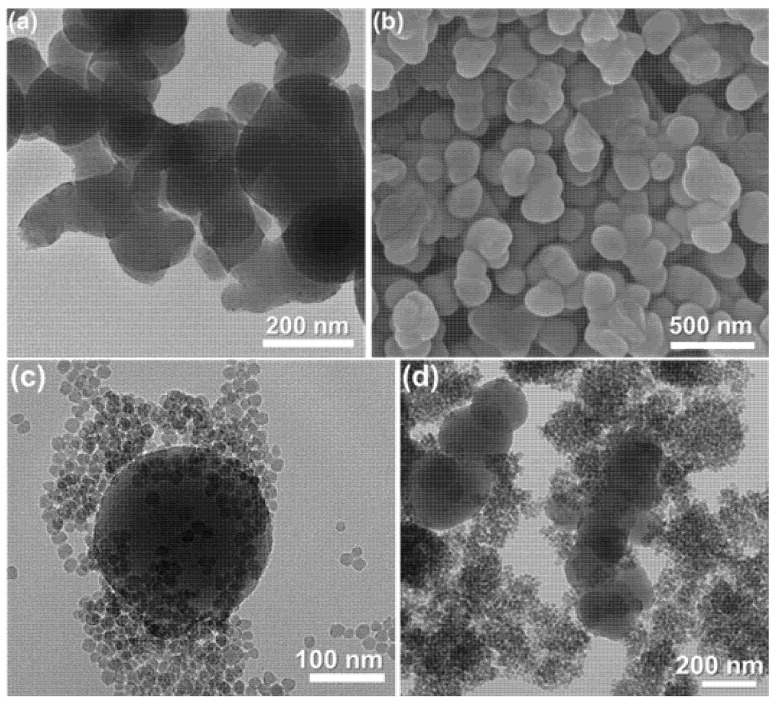
TEM (**a**) and FESEM (**b**) image of pristine porphyrin polymer (FePOP-1); (**c**,**d**) TEM image of PorPOPS.

**Figure 3:**
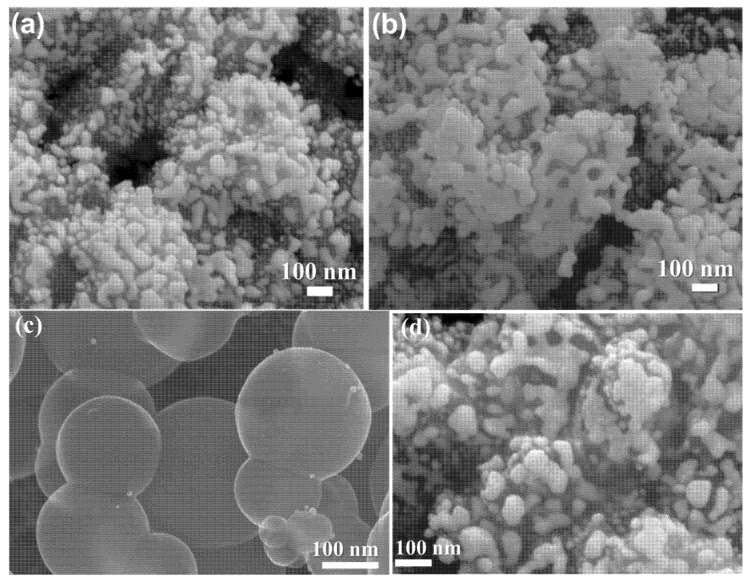
FESEM images of (**a**,**b**) sulfonated porphyrin silica composite, PorPOPS, (**c**) FePOP-1S, (**d**) un-functionalized PorPOP.

Morphology was confirmed from TEM and FESEM image analyses; it suggests an agglomerated nanostructure of PorPOPS and PorPOP, containing silica nanoparticles. It shows that 150–250 nm porphyrin polymers are surrounded by smaller silica spheres of about 30 nm (Figure 2 and Figure 3). The spherical shape of pristine (FePOP-1) and the sulfonated porphyrin nanoparticles (FePOP-1S) are evidenced from both TEM and SEM images, with a wider distribution of particles (150–300 nm).

**Figure 4:**
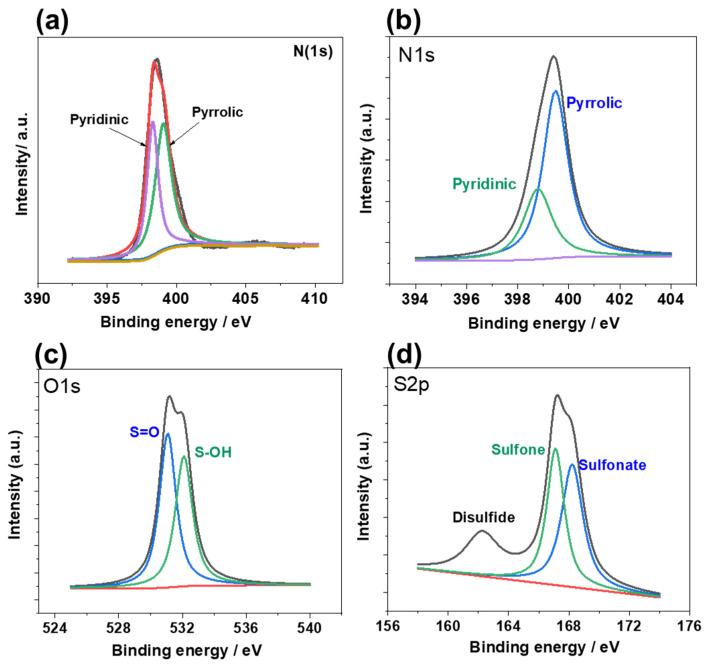
XPS spectra of (**a**) N1s of PorPOP and (**b**–**d**) PorPOPS containing N1s, O1s and S2p, respectively.

Chemical composition of the surface was assigned through X-ray photoelectron spectroscopic (XPS) analysis (Figure 4). Deconvoluted N1s spectrum in PorPOPS confirms the existence of pyrrolic and pyridinic moieties, centered at 399.8 and 398.9 eV, respectively [28]. However, XPS shows no existence of either oxidized nitrogen or graphitic nitrogen beyond 400 eV [29]. Thus, XPS infers the appearance of a large pyrrolic band corresponding to the porphyrin moiety of PorPOPS. Hence, this suggests that PorPOPS is stable under strong acid treatment. Interestingly, intensity of the pyridinic band is suppressed in PorPOPS in contrast to un-functionalized PorPOP, which might be because of the protonated porphyrin in PorPOPS. Successful grafting of sulfonic acid is further confirmed from the deconvoluted S2p spectrum. A fitted doublet appears at 167 and 168.5 eV and accompanies a broad shoulder at 162.2 eV. The strong band at 168.5 eV resembles a sulfonate band, while another band appearing at 167 eV could be attributed to oxidized sulfone (−SO_2_−). On the other hand, the broad shoulder may originate from organic disulfide-type residues [30]. Considering elemental analysis from XPS, higher pyrrolic (70%) to pyridinic (30%) ratio and a better sulfone (58%) to sulfonate (52%) ratio can be predicted. Furthermore, CHNS analysis reveals the possibility of ~5.0 wt% sulfonic acid sites in PorPOPS. Thus, the above results clearly demonstrate that a sulfonic-acid-rich porphyrin network is successfully formed in PorPOPS.

## 3. Discussion

**Scheme 2:**
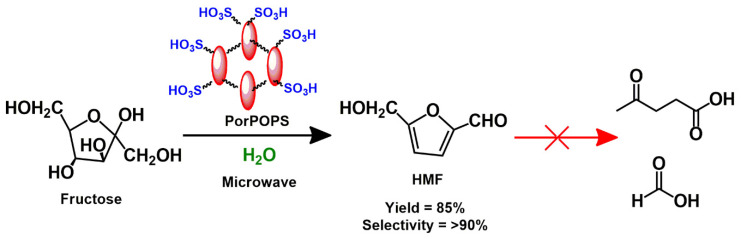
One-pot green conversion of fructose to HMF using PorPOPS.

A high surface area and the presence of accessible sulfonic acids in PorPOPS motivated us to explore its catalytic activity towards fructose dehydration reactions (Scheme 2).

**Figure 5:**
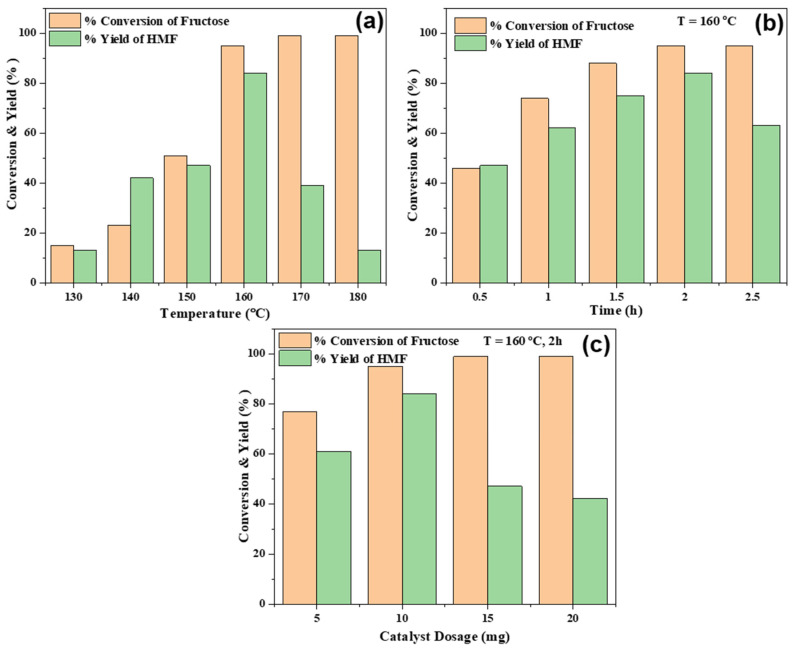
Effect of temperature (**a**), time (**b**) and catalyst dosage (**c**) for PorPOPS catalysed conversion of fructose to HMF in 2 mL water.

Initial screening of the reaction in a microwave reactor was done at different temperatures and times using various amounts of catalyst to optimize the reaction conditions. Contrary to the literature, we obtained the highest HMF yield in water; thus, all the reactions were carried out using water only as a solvent. The effect of temperature on the fructose conversion and the yield of HMF was thoroughly studied within 130 to 180 °C (Figure 5a). Initially, at 130 °C, only 15% fructose was converted to 13% HMF (yield). Further increases in temperature (from 130 to 160 °C) led to a significant improvement in both the conversion and yield, where highest HMF yield (85%) was observed at 160 °C. Although fructose conversion (>95%) progressively increased at higher temperatures (>160 °C), HMF yield was reduced from 85% (160 °C) to 39% (170 °C) and 13% (180 °C), which indicates fructose dehydration is highly sensitive to temperature, and an optimum temperature is necessary to maintain high HMF selectivity, as other side products (levulinic acid, formic acid) are commonly observed beyond 170 °C [31]. Again, polycondensation of fructose may also reduce HMF selectivity at 170 °C [32]. Thus, kinetic study was done at 160 °C, as shown in Figure 5b.

**Figure 6:**
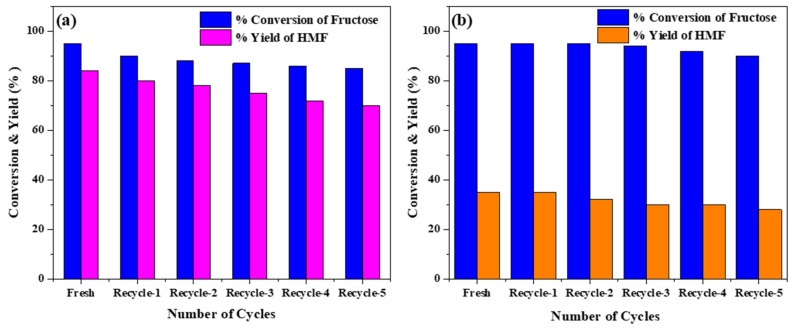
Recyclability study of (**a**) PorPOPS and (**b**) sulfonated porphyrin polymers (FePOP-1S) for fructose dehydration reaction to HMF (reaction conditions: 0.01 g fructose (0.04 mmol/g sulfonic acid), 2 mL water, 160 °C, 2 h).

At 0.5 h, lower conversion (46%) and yield (47%) were observed. Further prolonging the reaction to 2 h, HMF yield was improved to 85%, but decreased to 63% at 2.5 h (Figure 5b), possibly because of humin formation from fructose decomposition [33,34]. Thus, the controlled experiments suggest that 160 °C/2 h is the most suitable condition to achieve the highest HMF yield over PorPOPS. Since fructose dehydration is dependent on the total Brønsted acidity, variation of the catalyst dosage may change the reaction rate. For this, catalyst dosage was varied from 0.005 g (0.1 mmol/g sulfonic acid) to 0.02 g (0.4 mmol/g sulfonic acid); the highest yield of HMF occurred when 10 mg catalyst was used, which was subsequently reduced at higher loading, as shown in Figure 5c. Thus, a higher catalyst amount results in decomposition or degradation of fructose to other by-products. Recyclability of PorPOPS was tested up to five cycles; HMF yield was reduced to ~70% after the 5th cycle, in contrast to the fresh catalyst (85%) (Figure 6a). Additionally, the sulfonated porphyrin FePOP-1S was sustained up to five recycle runs, and ~14% loss of HMF yield was observed at the end of the 5th cycle. This result shows that sulfonated porphyrin with/without functionalization by silica nanoparticles is advantageous as a solid catalyst in water. Nevertheless, the un-functionalized PorPOP and FePOP-1 which do not have any sulfonic acid sites, cannot exhibit HMF yield (Table 1, Rows 13–14). However, due to feeble acidity of silica hydroxyl sites, only 5% fructose is converted with PorPOP. This result suggests that a cooperative interaction between porphyrin, silica and sulfonic acids in PorPOPS is essential for maximum conversion of fructose to HMF. A comparative study with other contemporary catalysts, e.g., porous carbon, zeolite, metal-organic frameworks (MOF) and metal oxides, is shown in Table 1.

Interestingly, PorPOPS shows highest HMF yield among the contemporary solid acid catalysts. It is noteworthy that stability could be an issue for MOFs in water, as shown by Corma and Boronat. Importantly, it was shown that high HMF yield in water can be achieved under anaerobic conditions in nitrogen (1 bar) atmosphere [43]. Interestingly, the effect of silica on PorPOPS is significant, where microporous sulfonated porphyrin polymer synthesized in the absence of colloidal silica showed lower HMF yield (35%) under similar conditions. Unlike sulfonated porphyrin POP, unique mesoporosity, together with hydrophilic coating of silica on the hydrophobic surface, may be advantageous for the activation and dehydration of fructose to HMF. Thus, for fructose conversion, PorPOPS is advantageous in water, possibly due to its high surface area and good accessibility of aromatic sulfonic acids towards substrates, as previously observed by Schüth et al. [44].

## 4. Materials and Methods

### 4.1. Chemicals

Pyrrole (≥98%), anhydrous FeCl_3_ (99%), glacial acetic acid (≥99%), chlorosulfonic acid (99%), terephthalaldehyde (99%), anhydrous 1,2-dichloroethane (99.8%), fructose (≥99%), 5-hydroxymethylfurfural (≥99%), sulfuric acid (99%), colloidal silica (Ludox, AS-40) and HPLC-grade water were purchased from Sigma Aldrich, St. Louis, MO, USA. All chemicals were used as received, without any further purification.

### 4.2. Instrumentation

N_2_ adsorption-desorption isotherms were measured at −196 °C using a Quantachrome Instruments (A brand of Anton Paar, Boynton Beach, Florida, USA) Autosorb 1C surface area analyzer. Prior to the analysis, samples were degassed at 150 °C for 5 h under high vacuum. Pore size was analyzed with a density functional theory (DFT) model using N_2_ at 77 K on carbon cylindrical pores. The thermal stability of the samples was measured by thermogravimetry analysis (TGA) using a NETZSCH STA 449F3 analyser (NETZSCH, Selb, Germany) within the temperature range 25–1000 °C at a heating rate of 10 °C/min in air. X-ray photoelectron spectroscopy (XPS) was recorded by VG ESCALAB MK2 (ThermoFisher Scientific, Waltham, MA, USA) using AlKα (hλ = 1486.6 eV) as the excitation source. Fourier transform infrared spectra (FT-IR) were documented on a Nicolet Nexus 470 IR spectrometer (ThermoFisher Scientific, Waltham, MA, USA). Field-emission scanning electron microscopy (JEOL JEM 6700F FE SEM, JEOL, Tokyo, Japan) was used to examine the morphology of powder samples. Transmission electron microscopy (TEM) experiments were executed using HITACHI HT7700 (HITACHI, Tokyo, Japan) at an acceleration voltage of 100 kV. Carbon, hydrogen and nitrogen contents were determined using a Perkin Elmer 2400 Series II CHN analyzer (Perkin Elmer, Waltham, MA, USA).

### 4.3. Synthesis of Organic-Inorganic Hybrid Silica-Porphyrin Conjugated Polymers (PorPOPs)

A porphyrin polymer (FePOP-1) was reproduced from our previous report after slight modification of the procedure [22]. Initially, terephthalaldehyde (0.2 g, 1.5 mmol) was taken in a 100 mL well dried beaker and was dissolved in 15 mL of glacial acetic acid under constant stirring for 10 min. Next, freshly distilled pyrrole (0.1 g; 1.5 mmol) was added to the solution and stirred for 20 min with the addition of 5 mL colloidal silica. Anhydrous FeCl_3_ (0.32 g; 2 mmol) was added to the solution, and stirring was continued to 12 h under N_2_ atmosphere. The brownish mixture was transferred to a Teflon-lined autoclave and was subjected to hydrothermal treatment at 180 °C for 48 h under a static condition. The dark brownish precipitate was then filtered and washed thoroughly with distilled water, methanol and THF to remove all iron-based impurities and then dried in an oven at 80 °C for 24 h (named as PorPOP). The yield of polymer was 25% based on terephthalaldehyde, and the elemental analysis (wt%) was C, 80.0; H, 4.1; N, 8.5.

### 4.4. Synthesis of PorPOPS Through Post-Functionalization with Chlorosulfonic Acid

A total of 0.3 g PorPOP was dispersed in 10 mL anhydrous 1,2-dichloromethane through sonication for 5 min. The slurry mixture was cooled in an ice bath, and chlorosulfonic acid (2 mL) was then added slowly to the solution and stirred for three days at 25 °C. After completion, the dark brownish solution was poured on ice, centrifuged, and washed thoroughly with water until pH was neutral. Then, the catalyst was dried in an oven overnight and named PorPOPS. Elemental analysis (wt%) was as follows: C, 30; H, 3.1; N, 5.2; S, 5. The total acid strength of PorPOPS was measured through acid-base titration and was 1.96 mmol/g.

Un-functionalized porphyrin polymer (FePOP-1) in absence of silica was reproduced from a previous report [22] and was post-functionalized with sulfonic acid following the procedure mentioned above. The sulfonic acid functionalized organic polymer was named Fe-POP-1S.

### 4.5. One-Pot Conversion of Fructose to HMF

All fructose dehydration reactions were performed using a microwave reactor (Anton Paar: Monowave 300, Anton Paar, Graz, Austria) with operation limits of 300 °C and 30 bar. In a typical reaction, 0.02 g fructose, PorPOPS (0.005 to 0.02 g) and 2 mL water was fed in the borosilicate glass vial. A magnetic bead was placed in the vial for stirring and the sealed vial was placed in the reactor cavity. Then, a series of reactions were performed under different temperatures (130 to 180 °C) for varying reaction times (0.5 h to 2.5 h) using the as-fast-as-possible heating mode of the microwave reactor [45]. After the completion of the reaction, the reactor was cooled down to room temperature using the cooling system provided with the reactor. All the reactions were performed at least twice, and the average of the results were reported.

### 4.6. Product Analysis

After the reaction, the catalyst was separated from the liquid product mixture using filtration. Then, the liquid samples were passed through a micro-syringe filter before analysing the samples using high-performance liquid chromatography (HPLC). HPLC (Agilent 1200 infinity series (Agilent Technologies, Santa Clara, CA, USA) equipped with an autosampler, refractive index (RI) detector, ultraviolet (UV) detector, and Aminex HPX-87H column (Bio-Rad Laboratories, Irvine, CA, USA) (300 × 7.8mm) was used for the quantification of fructose and HMF. An injection volume of 2 µL and flow rate of 0.6 mL/min were fixed, and sulphuric acid (5 mM) was used as the mobile phase. The column temperature was maintained at 50 °C, and the UV detector was set at a wavelength of 284 nm for the detection of HMF. All the samples were injected twice, and the average readings were used for the calculation of the fructose conversion and the calculation of HMF yield, using the following equations (Equations (1) and (2)), based on their mass in the feed:

(1) $$\%\;\mathrm{Conversion}\;\mathrm{of}\;\mathrm{fructose}=\frac{\mathrm{initial}\;\mathrm{amount}\;\mathrm{of}\;\mathrm{fructose}\;\mathrm{fed}-\mathrm{amount}\;\mathrm{of}\;\mathrm{unreacted}\;\mathrm{fructose}}{\mathrm{initial}\;\mathrm{amount}\;\mathrm{of}\;\mathrm{fructose}\;\mathrm{fed}}\times 100$$

(2) $$\%\;\mathrm{Yield}\;\mathrm{of}\;\mathrm{HMF}=\frac{\mathrm{amount}\;\mathrm{of}\;\mathrm{HMF}\;\mathrm{produced}}{\mathrm{initial}\;\mathrm{amount}\;\mathrm{of}\;\mathrm{fructose}\;\mathrm{fed}}\times 100$$

## 5. Conclusions

Experimental results shown herein suggest that sulfonated porous porphyrin polymer impregnated with silica nanoparticles is an efficient solid acid catalyst for one-pot conversion of biomass-derived fructose to HMF. Remarkably, HMF yield (85%) in water is higher than that reported over related solid acid catalysts. This could be due to its high porosity accompanied by high loading of accessible surface sulfonic acid groups at the catalyst surface. Although aromatic sulfonates are demanding in acid catalysis, desulfonation and humin formation is a problematic issue for biomass conversion at high temperature reactions. Sulfonic acid functionalized POPs with silica support can afford excellent yield of HMF from biomass-derived carbohydrates, and there is a huge opportunity to scale up this conversion beyond the laboratory.

## 6. Patents

An Indian Patent for the work reported in this manuscript has been filed (Application No.: 202111012815).

## Figures and Tables

**Table 1:** Comparative study of PorPOPS in fructose conversion to HMF in water.

Entry	Reaction Conditions	Catalyst	Conversion of Fructose (%)	Yield of HMF (%)	Reference
1	120 °C, 16 h	Phosphorylated mesoporous carbon	78	41.3	[35]
2	100 °C, 3 h	Niobium phosphate	51.2	30.4	[36]
3	180 °C, 10 min	Niobium phosphate	86.5	33.9	[37]
4	190 °C, 8 min	Zirconium phosphate	96.3	39.4	
5	170 °C, 4.5 h	Phosphoric acid functionalized carbon catalyst	100	59.9	[38]
6	120 °C, 24 h	Amberlyst-15	57	15	[39]
7	165 °C, 3 h	TiO_2_-SO_3_H	99	50	[40]
8	140 °C, 5 h	NU-1000	60	2.3	[41]
9	140 °C, 5 h	PO_4_/NU(half)	50	15	
10	120 °C, 1 h	SBA-15-SO_3_H	100	30	[42]
11	160 °C, 2 h	Sulfonated porphyrin polymer (FePOP-1S)	95	35	This study
12	160 °C, 2 h	PorPOPS	95	85	This study
13	160 °C, 2 h	Un-functionalized PorPOP	<5	traces (<1)	This study
14	160 °C, 2 h	Un-functionalized FePOP-1	No	No	This study

## Data Availability

Data are available from the authors on request.

## Sample Availability

Samples of the compounds are available from the authors.
